# Radiation damage by extensive local water ionization from two-step electron-transfer-mediated decay of solvated ions

**DOI:** 10.1038/s41557-023-01302-1

**Published:** 2023-08-24

**Authors:** G. Gopakumar, I. Unger, P. Slavíček, U. Hergenhahn, G. Öhrwall, S. Malerz, D. Céolin, F. Trinter, B. Winter, I. Wilkinson, C. Caleman, E. Muchová, O. Björneholm

**Affiliations:** 1 Department of Physics and Astronomy, Uppsala University, Uppsala, Sweden; 2 FS-BIG, DESY, Hamburg, Germany; 3 Department of Physical Chemistry, University of Chemistry and Technology, Prague, Czech Republic; 4 Fritz-Haber-Institut der Max-Planck-Gesellschaft, Berlin, Germany; 5 MAX IV Laboratory, Lund University, Lund, Sweden; 6 Synchrotron SOLEIL, L’Orme des Merisiers, Saint-Aubin, Paris, France; 7 Institut für Kernphysik, Goethe-Universität Frankfurt am Main, Frankfurt am Main, Germany; 8 Institute for Electronic Structure Dynamics, Helmholtz-Zentrum Berlin für Materialien und Energie, Berlin, Germany; 9 Center for Free-Electron Laser Science, DESY, Hamburg, Germany

**Keywords:** Photochemistry, Electron transfer

## Abstract

Biomolecular radiation damage is largely mediated by radicals and low-energy electrons formed by water ionization rather than by direct ionization of biomolecules. It was speculated that such an extensive, localized water ionization can be caused by ultrafast processes following excitation by core-level ionization of hydrated metal ions. In this model, ions relax via a cascade of local Auger–Meitner and, importantly, non-local charge- and energy-transfer processes involving the water environment. Here, we experimentally and theoretically show that, for solvated paradigmatic intermediate-mass Al^3+^ ions, electronic relaxation involves two sequential solute–solvent electron transfer-mediated decay processes. The electron transfer-mediated decay steps correspond to sequential relaxation from Al^5+^ to Al^3+^ accompanied by formation of four ionized water molecules and two low-energy electrons. Such charge multiplication and the generated highly reactive species are expected to initiate cascades of radical reactions.

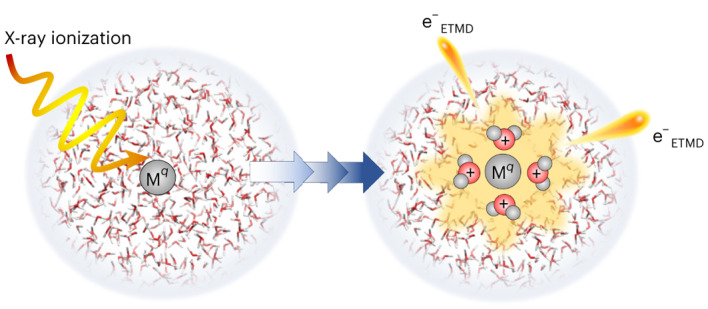

## Abstract (web summary)

Radiation damage in biology is largely mediated by radicals and low-energy electrons formed by water ionization and extensive, localized water ionization can be caused by ultrafast processes following the core-level ionization of hydrated metal ions. Now it has been shown that, for Al^3+^ ions, relaxation occurs via sequential solute–solvent electron transfer-mediated decay.

## Main

**Fig. 1:**
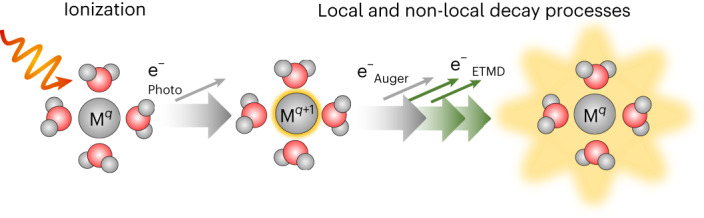
Sketch of the processes that occur following core-level ionization. Ionization of solvated metal ions, M^*q*^, produces M^*q*+1^ ions, which undergo a local Auger–Meitner decay but in many cases cannot further decay locally. In water, the ions are destabilised by the environment and may undergo ETMD, extensively ionizing surrounding water molecules.

**Extended Data Fig. 1:**
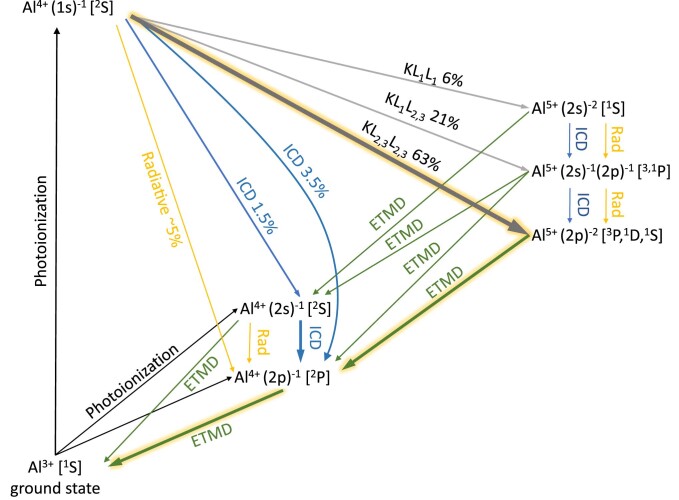
Schematic overview of possible electronic decay processes of aqueous Al^3+^. Schematic overview of the electronic decay processes that can be considered after 1s core ionization of aqueous Al^3+^. After 1s ionization, the dominant KLL Auger-Meitner decay process leads to highly charged Al^5+^ states. Minor channels after 1s ionization are ICD (blue lines) and X-ray fluorescence (radiative, orange arrows). The highly charged Al^5+^ states with high excess energy can decay by ICD (blue lines). The states with less excess energy undergo ETMD processes (green lines). The cascades continue until the highly ionized cations are reduced to the original Al^3+^ state. The cascade studied in this work is highlighted in yellow.

Radiation damage, particularly radiolysis of water and aqueous solutions, is typically considered to take place randomly along the radiation path. Low-energy electrons and free radicals, formed in the surrounding aqueous medium, reach biomolecules by diffusion and cause damage^1–3^. Metal atoms, which are crucial for the function of many biomolecules^4^, have been theoretically predicted (exemplified for Mg^2+^ in water) to form local centres for radiation damage, on the timescale of a few hundred femtoseconds, via a cascade of local and non-local decay processes (see the simplified decay scheme in Fig. 1). Note that the overall predicted cascade of ultrafast decay processes^5^ is more complex; all relevant processes are shown in Extended Data Fig. 1.

In this Article, we experimentally confirm the occurrence of a multi-step, charge-multiplying relaxation mechanism for Al^3+^ in water. We identify an important sequential non-local decay path, whereby core ionization of the metal ions in aqueous solution leads to a concerted formation of slow electrons and several water ions, which will develop into radicals within 1 ps (refs. ^6,7^).

The first step of radiation damage is ionization, and, for the photon energies relevant for X-ray applications, the principal ionization channel is core-level ionization. Biomaterials primarily consist of low-*Z* atoms, but high-*Z* atoms contribute more to radiation damage than their concentration suggests, due to their higher ionization cross-sections. Following core ionization of isolated molecules or atoms, rapid Auger–Meitner decay leads to multiply ionized states within just a few femtoseconds, from which the only de-excitation channel is photon emission, occurring on a much longer timescale. The situation changes if the excited species are embedded in an environment that enables electronic de-excitation via energy transfer between neighbouring molecules (for example, in liquid water). Such considerations led to the prediction, more than two decades ago, of non-local electronic decay processes, most prominently intermolecular Coulombic decay (ICD) and electron transfer-mediated decay (ETMD)^8,9^. Since its prediction, ICD has been observed in a plethora of systems ranging from dimers^10–15^ to clusters^16–18^ and aqueous solutions^19–22^ (for a recent review, see ref. ^23^). ICD has also been predicted to play a role in the repair of DNA molecules^24^. Furthermore, two research groups simultaneously succeeded in observing single-step ETMD in noble-gas dimers and clusters^25,26^. Since then, similar single-step processes were also observed in liquids^27,28^.

**Fig. 2:**
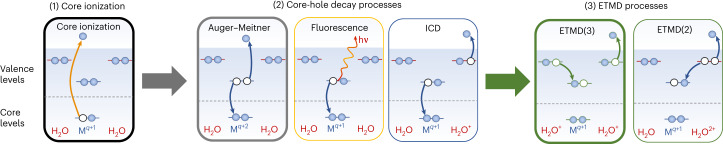
Schematic illustration of the electronic transitions and resulting states induced by core-level ionization of a solvated metal ion, M^*q*^, with an initial charge *q*. Ionization of the core level results in an M^*q*+1^ ion (first step). The core-hole decay processes (second step) include two local phenomena, Auger–Meitner and fluorescence decay, and a non-local channel, ICD. In the dominant Auger–Meitner decay, the core hole is filled by a metal-ion valence electron, with the released energy leading to emission of another metal-ion valence electron, resulting in M^*q*+2^. In the fluorescence decay, the energy released is emitted as a photon, resulting in M^*q*+1^. ICD constitutes a process in which the released energy leads to emission of a valence electron from a neighbouring species, resulting in M^*q*+1^ and H_2_O^+^. In a third step, the metal outer-valence holes produced in the second step can be filled via different ETMD processes. Assuming the second step took place by Auger–Meitner decay, in the ETMD(2) process, a valence hole on M^*q*+2^ is filled by a valence electron from a neighbouring water molecule, and the released energy causes emission of another valence electron from the same water molecule, resulting in M^*q*+1^ and H_2_O^2+^. In ETMD(3), a valence hole on M^*q*+2^ is filled by a valence electron from a neighbouring water molecule, and the released energy causes emission of another valence electron from yet another water molecule, resulting in M^*q*+1^ + 2(H_2_O^+^). The remaining valence hole on the metal centre can be filled in another ETMD process (not shown).

ICD and ETMD take on greater importance when multi-step decay cascades are considered^5,29,30^. As a concrete example, we consider core-level ionization of a metal ion M^*q*^ of initial charge *q*, solvated in water. All of the aforementioned secondary-electron emission processes following core ionization—Auger–Meitner decay, ICD and ETMD—involve two active electrons, one filling an inner-valence or core-level hole and one being ejected, as schematically shown in Fig. 2. For local Auger–Meitner decay, both active electrons are associated with the core-ionized species, resulting in M^*q*+2^. This contrasts with the two non-local decay mechanisms; ICD involves one electron on the core-ionized species and one from a neighbouring species, that is, a water molecule, while in ETMD, both active electrons originate from neighbouring species. In the ETMD process, the emitted electron can originate from the same water molecule that donated an electron to the excited metal ion. This process is denoted ETMD(2) in ref. ^31^, as it involves only two species: the metal ion and one water molecule. An example of an Auger–Meitner process followed by ETMD(2) decay is shown in Fig. 2; in the ETMD(2) step, M^*q*+1^ + H_2_O^2+^ species are produced. Alternatively, an ETMD electron can be emitted from another water molecule in close proximity, via a process referred to as ETMD(3) in ref. ^31^, as it involves three species, the metal ion and two different hydrating water molecules. In the Fig. 2 example, this produces a M^*q*+1^ + 2(H_2_O^+^) final state. In this text, for the sake of simplicity, we will limit further discussion to the ETMD(3) channels, which have lower energy according to the ab initio calculations, due to the energetically favourable hole delocalization. It is important to mention, however, that for the current case, neither experimental data nor theoretical calculations can provide a decisive answer about the prevalence of ETMD(2) or ETMD(3). In previous work^27^, ETMD(3) was found to dominate over ETMD(2). However, such results cannot be simply applied to the current case.

In general, following X-ray irradiation, local Auger–Meitner decay dominates over alternative relaxation processes if it is energetically allowed. For the non-local decays, ICD is more prominent than ETMD, whenever the former is energetically feasible^23^. Both Auger–Meitner and ICD transitions can terminate in excited states that have sufficient energy for further electronic decay but lack electrons in the higher-lying shells to refill any remaining photo-generated electron vacancies and lower the system’s energy. At this point, ETMD gains importance, since it does not rely on high-lying electrons in the excited species itself to refill the vacancies and, for light elements, still occurs on a substantially faster timescale than fluorescence^5,32^.

**Fig. 3:**
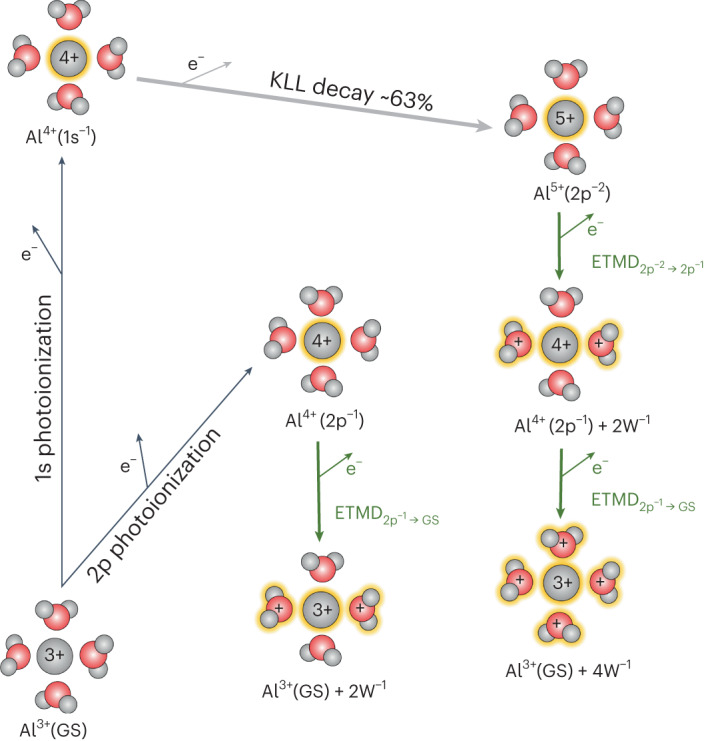
Schematic of the investigated processes and states generated after 1s ionization of solvated Al^3+^. The first step forms Al^4+^(1s^−1^) ions and initiates a decay cascade, where ~90% of the ionized species undergo local KLL Auger–Meitner decays to predominantly (~63%) form Al^5+^(2p^−2^) states. Alternatively, a single Al 2p level vacancy can be created by initial ionization at lower photon energy (2p^−1^). These 2p vacancies are filled by non-local ETMD processes, in the case of Al^4+^(2p^−1^) in one ETMD_2p−1→GS_ step, and for Al^5+^(2p^−2^) in two steps, ETMD_2p−2→2p−1_ followed by ETMD_2p−1→GS_, after which the Al ion is back to its ground state Al^3+^ (GS). Each ETMD process ionizes up to two water molecules and emits one slow ETMD electron. Note that, for clarity, the figure only shows ETMD processes involving two water molecules, ETMD(3); for further details, see the main body of text.

X-ray-induced, non-local secondary-electron emission processes and the local formation of multiple ionized water molecules have been discussed for the exemplary case of solvated Mg^2+^ metal ions^5^. There, ab initio calculations predicted that core ionization, followed by local Auger–Meitner decay and subsequent non-local ICD and ETMD processes, leads to an extensive formation of radicals and slow electrons in the vicinity of the metal ion, within just a few hundred femtoseconds. To test these predictions, we have investigated the multi-step electronic relaxation processes that occur following X-ray irradiation of aqueous Al^3+^ ions (for further details, see Extended Data Fig. 1), which are isoelectronic with Mg^2+^. Here, we focus on the non-local relaxation of the aqueous-phase Al^4+^ and Al^5+^ ions, produced by core-level ionization and the main subsequent relaxation, local KLL (the primary hole in the K shell, and the two final state holes in the L shell) Auger–Meitner decay to the Al^5+^(2p^−2^) configuration. The corresponding electronic transitions and processes occurring in our experiments are sketched in Fig. 3.

Starting from the ground state of Al^3+^, with a 1s^2^2s^2^2p^6^ electronic configuration, an Al^4+^(1s^−1^) state is created by photoionization. This state relaxes via KLL Auger–Meitner decay in ~90% of the cases^33^. (To simplify the discussion, we reduce the amount of Auger–Meitner decay found in the atomic calculation of ref. ^33^ by the percentage of ICD relative to the Auger–Meitner channel in aqueous Al^3+^, as deduced in ref. ^34^.) The KLL Auger–Meitner decay can result in different electronic configurations: 2s^−2^, 2s^−1^2p^−1^, or 2p^−2^. The branching ratios between these three state configurations have not been determined for Al^3+^ in water. Furthermore, interpretation of data for metallic Al is complicated by the presence of 3s and 3p electron signatures, as well as extensive plasmon satellite peak formation. However, for the isoelectronic Ne atom, the branching ratios of the KLL Auger–Meitner state configurations were determined as ~7% for 2s^−2^, ~23% for 2s^−1^2p^−1^, and ~70% for 2p^−2^ (refs. ^35,36^). We expect a similar branching ratio for Al^3+^ in water (taking into account the amount of Auger–Meitner decay of 90%): ~6% for 2s^−2^, ~21% for 2s^−1^2p^−1^, and ~63% for 2p^−2^. We first focus on the major Al^4+^(1s^−1^) → Al^5+^(2p^−2^) + e^−^ decay channel, before briefly returning to the other channels. We will notably neglect double Auger decays here, which have been shown to account for ~5.7% of the 1s^−1^ state decays in isoelectronic Ne atoms^37^.

The Al^5+^(2p^−2^) state cannot locally relax, due to a lack of electrons in higher-lying energy levels of the Al ion. Correspondingly, the next decay step has to involve electrons from the surrounding water molecules via non-local processes, such as radiative charge transfer or ETMD^5,38^. In such a case, ETMD is expected to dominate, if energetically allowed^38^. Generally, simple energy considerations are helpful as a first indication whether ETMD—that is, the conversion of one Al 2p vacancy into two water valence vacancies and an outgoing electron—is possible. In the aqueous Al^3+^ case, the lowest binding energies of the water molecules in the first solvation shell are 11.97 eV (ref. ^34^), and the binding energy of Al 2p in water is 80.4 eV, that is, much more than twice the hydration-shell–water binding energy. ETMD, involving electrons from the surrounding water molecules, is thus energetically allowed.

The ETMD processes that are investigated here are sketched in Fig. 3. We will distinguish between the ETMD processes from Al^5+^(2p^−2^) to Al^4+^(2p^−1^), ETMD_2p−2→2p−1_, and from Al^4+^(2p^−1^) to the ground state Al^3+^(GS), ETMD_2p−1→GS_. After 1s ionization and subsequent KLL Auger–Meitner decay to Al^5+^(2p^−2^), one of the Al ion 2p holes is filled by an electron from a neighbouring water molecule in the first ETMD_2p−2→2p−1_ step, with another electron being emitted from water: Al^5+^(2p^−2^) + 2W → Al^4+^(2p^−1^) + 2W^−1^ + e^−^, where W^−1^ denotes a valence vacancy on a water molecule (W). This transition results in the generation of two water molecule vacancies and the reduction of the Al charge to 4+.

The remaining 2p^−1^ hole formed in the first ETMD_2p−2→2p−1_ step can decay in a second step, ETMD_2p−1→GS_: Al^4+^(2p^−1^) + 2W^−1^ + 2W → Al^3+^(GS) + 4W^−1^ + e^−^. By that the initially core-ionized aluminium ion is returned to its original 3+ charge state. Together, the two ETMD steps thus produce a total of four water ions and two emitted electrons.

Concerning alternative and minor core-hole relaxation pathways, about 5% of the generated Al^4+^(1s^−1^) states do not relax via KLL Auger–Meitner decay^33^, but by Kα fluorescence decay, Al^4+^(1s^−1^) → Al^4+^(2p^−1^) + *γ*. Just like the Al^5+^(2p^−2^) state discussed in the previous paragraph, the Al^4+^(2p^−1^) state cannot undergo further local relaxation processes due to the lack of higher-lying electrons in the Al^4+^ ions. It will alternatively relax in an ETMD_2p−1→GS_ process, similar to the second ETMD step: Al^4+^(2p^−1^) + 2W → Al^3+^(GS) + 2W^−1^ + e^−^. As we will discuss in the last part of the ‘Results and discussion’ section, we can selectively probe the ETMD_2p−1→GS_ process by initially ionizing the Al^3+^ 2p level (instead of the Al^3+^ 1s level) at lower photon energies.

In addition to the two local core-hole decay processes, KLL Auger–Meitner decay and Kα fluorescence decay, about 5% of the generated Al^4+^(1s^−1^) states decay via non-local ICD processes, resulting in Al^4+^(2p^−1^) + W^−1^ (ref. ^34^). The 2p hole may then relax in an ETMD_2p−1→GS_ process, Al^4+^(2p^−1^) + W^−1^ + 2W → Al^3+^(GS) + 3W^−1^ + e^−^ (Extended Data Fig. 1).

Generally, inferences can be made about ETMD decay mechanisms by performing X-ray spectroscopy experiments and such mechanisms can be validated by means of high-level ab initio calculations. Theory can in principle access the energetics of different electronic configurations of the solvated Al ions that are created during the different ETMD steps introduced in the previous paragraphs. Furthermore, it can identify the electron kinetic energy regions in which the first and second ETMD steps, following 1s ionization and local Auger–Meitner decay, should occur. However, the individual electronic relaxation steps involve highly ionized states, with charges distributed over neighbouring molecules and with strong interactions with the environment. These calculations thus present a challenge even for state-of-the-art electronic-structure theory and spectral simulations.

We have applied advanced liquid-jet photoemission spectroscopy and spectral simulation methods to interrogate the photoionization, local Auger–Meitner decay, and non-local relaxation phenomena that occur following K-shell ionization of aqueous Al^3+^ ions. Based on the associated results and analysis, we address the following four questions here: (1) Does ETMD occur after the generation of Al^5+^(2p^−2^) and/or Al^4+^(2p^−1^) states? (2) Does a non-local-ionization cascade occur after metal-ion 1s ionization? (3) Which species are formed in the relaxation cascades? (4) What are the consequences of the exposed relaxation mechanisms for radiation chemistry?

## Results and discussion

### Experimental fingerprints of the ETMD cascade

A first, rough estimate of the kinetic energies of the electrons emitted in the first ETMD_2p−2→2p−1_ and second ETMD_2p−1→GS_ steps following 1s ionization can be obtained based on differences between the experimentally determined energies of the electronic levels involved in the decay (Extended Data Table 1). By assuming the Coulomb penalty energy (*E*_CP_) to be zero, the upper limits of the kinetic energy of the first and second ETMD steps are obtained as 82.5 eV and 58.6 eV, respectively. The Coulomb penalty is the energy resulting from having two positively charged ions in close proximity.

**Fig. 4:**
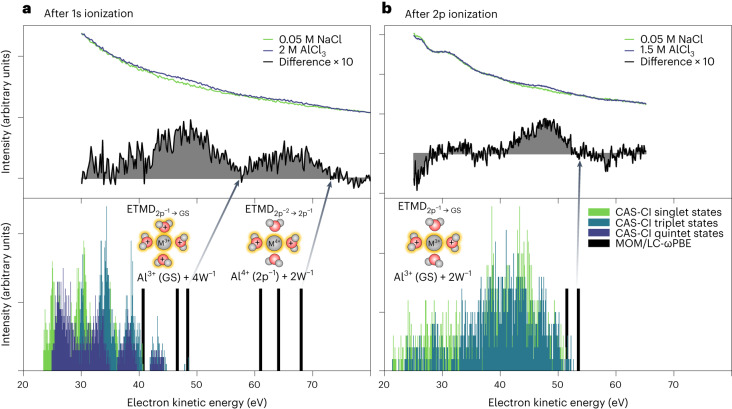
Comparison between experimental electron spectra in the ETMD region and ab initio calculations of the respective transition energies. **a**,**b**, Top: the electron spectra recorded in the region of the ETMD signals (blue) after 1s ionization (**a**) and 2p ionization (**b**), together with the respective background measurements (green) and the resulting difference spectra (grey). Bottom: the calculated density of the ETMD final states after 1s (**a**) and 2p (**b**) ionization. The energies were calculated at the CAS-CI/cc-pVDZ level. The states were shifted so that the lowest-energy state corresponds to the LC-*ω*PBE/aug-cc-pVTZ and aug-cc-pCVTZ value obtained in the polarizable continuum. The black bars show the highest kinetic energies for the first and second ETMD steps obtained at the MOM/LC-*ω*PBE/aug-cc-pVTZ and aug-cc-pCVTZ level for [Al(H_2_O)_4_]^3+^ (corresponding to the onset of the experimental spectra (black arrows)). The coloured histograms highlight the electron kinetic energies associated with the CAS-CI states of a given multiplicity. Source data

Figure 4 (top panels) shows the experimentally obtained electron spectra in the kinetic-energy region where the ETMD signals are expected (blue spectra). This energy region is dominated by the low-energy tail, characteristic for photoemission spectra from condensed matter^39^. This results in the observed large structureless background signal of inelastically scattered electrons. The top panels in Fig. 4a,b show spectra obtained from the AlCl_3_ solution with different photon energies. In Fig. 4a (top), the spectra measured with a ~1,570 eV photon energy and above the Al 1s binding energy are shown. This photon energy enables the two cascaded steps, ETMD_2p−2→2p−1_ and ETMD_2p−1→GS_, illustrated to the right side of Fig. 3. The ETMD features are best presented as the difference spectrum, solution spectrum (blue) minus water reference spectrum (green), as shown in grey underneath the experimental spectra. The validity of such a water-reference subtraction, and hence the significance of the obtained ETMD spectral contributions, has been discussed in Ref. ^27^. It is noted that the low-energy tail of the neat-water photoemission spectrum is structureless for the photon energies used in the present study, ^27,28,39^ with any deviations arising from solute contributions.

As we expected, based on the considerations around Fig. 3, two distinct broad spectral features, near ~48 eV and ~66 eV kinetic energy, are observable. These features are indicative of the two consecutive ETMD steps, refilling the Al 2p orbitals with electrons from surrounding water molecules. We note that we performed analogous measurements for MgCl_2_ solutions, without observing any clear Mg^2+^ ETMD signal. This may be due to the lower kinetic energy of the Mg ETMD electrons, making them more difficult to identify on the high background signal of inelastically scattered electrons. Electron–electron coincidence measurements would notably have a larger sensitivity to the ETMD electrons, in this case^28^.

### Understanding the ETMD cascade via ab initio modelling

To obtain a deeper understanding of the two consecutive ETMD steps following 1s ionization, a more advanced theory based on structural sampling and ab initio calculations is required. The energies of various electronic states, corresponding to their minimum-energy edges, were obtained by the maximum-overlap method (MOM) (LC-*ω*PBE levels) and are collected in Table 1 (more associated data is presented in Extended Data Table 1), together with the experimentally determined electronic state and kinetic energy values. As can be seen from the table, by comparison with the experimentally determined energies, the absolute energies calculated for the two cluster types contain substantial errors, on the order of several electron volts. The error is particularly large for core-ionized states, which is primarily due to the neglect of relativistic effects. We have observed similar errors for solvated Na^+^, Mg^2+^, and Al^3+^ cations in ref. ^34^; a related, detailed discussion can be found in ref. ^40^. The differences between the results for the two cluster sizes are also remarkable; the differences approach 10 eV (see, for example, the energies of the final 4W^−1^ states). However, the estimated ETMD kinetic energies are calculated as energy differences, and we assume that a large part of the absolute energy errors cancels out, resulting in a maximal error of the estimated kinetic energies of just a few electron volts.

**Table 1:** Energetics of the relevant electronic states and decay transitions

Energy of state/decay	exp Al${}_{{\mathrm{aq}}}^{3+}$	[Al(H_2_O)_4_]^3+^	[Al(H_2_O)_6_]^3+^
*E*(1s^−1^)	1,567.7(4)	1,566.0	1,562.9
KE(KLL Auger–Meitner)	1,380.9(4)	1,381.5	1,371.9
KE(ETMD_2p−2→2p−1_)	~60–70		
*E*(2p^−2^) − *E*(2p^−1^ + 2W^−1^1b_1_1b_1_)		68.0	68.3
*E*(2p^−2^) − *E*(2p^−1^ + 2W^−1^1b_1_3a_1_)		64.1	–^*^
*E*(2p^−2^) − *E*(2p^−1^ + 2W^−1^3a_1_3a_1_)		61.0	–^*^
KE(ETMD_2p−1→GS_)	~40–55		
*E*(2p^−1^ + 2W^−1^) − *E*(4W^−1^(Q))		48.4	48.4
*E*(2p^−1^ + 2W^−1^) − *E*(4W^−1^(T))		44.6	47.8
*E*(2p^−1^ + 2W^−1^) − *E*(4W^−1^(S))		40.7	42.6
*E*(2p^−1^)	80.4(2)	83.8	80.7
KE(ETMD_2p−1→GS_)	~40–55		
*E*(2p^−1^) − *E*(2W^−1^(T))		53.5	53.2
*E*(2p^−1^) − *E*(2W^−1^(S))		51.5	51.6

Average energies (*E*) and energy differences, and electron kinetic energies (KE) corresponding to the transitions shown in Fig. 3. The experimentally determined excited state and KLL Auger–Meitner kinetic energies (first column) are reported following ref. ^34^. Experimentally determined energies are denoted in the exp Al${}_{{\mathrm{aq}}}^{3+}$ column. The calculations were performed for 20 structures of [Al(H_2_O)_4_]^3+^ and [Al(H_2_O)_6_]^3+^ clusters in a polarizable continuum at the MOM/LC-*ω*PBE level with an aug-cc-pVTZ basis set and aug-cc-pCVTZ on aluminium. Q (quintet), T (triplet), and S (singlet) denote multiplicities of final states. ^*^It was not possible to obtain the energies due to poor convergence of the wave function.

We first consider the first ETMD_2p−2→2p−1_ step: Al^5+^(2p^−2^) + 2W → Al^4+^(2p^−1^) + 2W^−1^ + e^−^, corresponding to the high-energy feature in Fig. 4a. The ETMD electron kinetic energies depend on which water orbitals the electrons came from. The lowest-energy 2W^−1^ state corresponds to both holes being associated with water 1b_1_ molecular orbitals. This results in ETMD electron kinetic energies of 68.0 eV for [Al(H_2_O)_4_]^3+^ and 68.3 eV for [Al(H_2_O)_6_]^3+^. These values represent the high-energy tail of the first ETMD_2p−2→2p−1_ feature, that is, the onset of the spectrum, which is experimentally determined to occur at ~71 eV. If deeper orbitals of water (for example, 3a_1_ type) are ionized in the first ETMD_2p−2→2p−1_ step, the resulting electron kinetic energies are shifted to lower energies. In the case of the smaller cluster model, [Al(H_2_O)_4_]^3+^, the predicted energies were 64.1 eV and 61.0 eV. Such calculated energies are also shown in Fig. 4a (bottom), as black bars. These energies match the higher-kinetic-energy ETMD_2p−2→2p−1_ spectral features, and we can, thus, conclude that the calculations support the suggested assignment.

The calculated energies corresponding to the second ETMD_2p−1→GS_ step: Al^4+^(2p^−1^) + 2W^−1^ + 2W → Al^3+^(GS) + 4W^−1^ + e^−^, lie between 40.7 eV and 48.4 eV for [Al(H_2_O)_4_]^3+^ and 42.6 eV and 48.4 eV for [Al(H_2_O)_6_]^3+^ for final states with various multiplicities. These values are again in agreement with the high-energy tail of the low-kinetic-energy feature in the experimental spectrum shown in Fig. 4a (top). Since the 4W^−1^ final state comprises four ionized water molecules, where all associated holes are localized in the valence orbitals, we were also able to estimate the energies of the higher-energy states via the Complete Active Space Configuration Interaction (CAS-CI) approach. We show the energies for the 20 considered structures of [Al(H_2_O)_4_]^3+^ in the form of coloured histograms, to present the density of available final states. These results differ in terms of which orbitals the electrons come from, the coupling between the holes, and the magnitude of *E*_CP_. As can be inferred from Fig. 4, the estimated energies of the final states span more than 20 eV, giving rise to a broad distribution of possible electron kinetic energies between 25 eV and 45 eV, in agreement with the experiment. Due to the exchange energy^41^, the singlet final states have higher energies than states with higher multiplicities, that is, the resulting kinetic energies are lower for transitions to singlet final states.

The cascade of ultrafast processes depicted in Extended Data Fig. 1 contains a variety of alternative pathways, which must be discussed to assess their possible contribution to the measured signal. We discuss the energetics and probabilities of these processes in the Supplementary Information.

### Inner-valence ionization and ETMD

Having discussed the ETMD processes after 1s ionization, we turn our attention to the simpler, single-step ETMD process occurring following 2p ionization. The single-step process is representative of the ~5% of the Al^4+^ 1s^−1^ states that undergo Kα fluorescence decay, instead of KLL Auger–Meitner decay. We reiterate that direct 2p ionization is also a minor channel at the photon energy used for 1s ionization, which is correspondingly ignored here, due to its low probability relative to 1s ionization^42^. For photon energies below the 1s binding energy, however, this channel becomes dominant, and nearer to its respective ionization edge it has a larger cross-section. The electron spectra associated with the ETMD_2p−1→GS_ process: Al^4+^(2p^−1^) + 2W → Al^3+^(GS) + 2W^−1^ + e^−^, are shown in Fig. 4b (top). The experimental spectrum consists of a single feature, which peaks at ~48 eV and extends up to a ~52 eV kinetic energy. The binding energy of the 2p electron is 80.4 eV, which, in accord with the simple model and considering *E*_CP_ = 0, results in a maximum kinetic energy of ~58 eV. The calculated maximum kinetic energies for the ETMD_2p−1→GS_ process following 2p ionization are slightly above 50 eV (Table 1). These kinetic energies and the final-state distribution are correspondingly found to be in good agreement with the experiments, as shown in Fig. 4b (bottom).

Both the second ETMD step following 1s ionization and the ETMD process after 2p ionization consist of the filling of one 2p hole on the aluminium ion. The two ETMD_2p−1→GS_ processes are, however, not identical: the second ETMD step following 1s ionization is Al^4+^(2p^−1^) + 2W^−1^ + 2W → Al^3+^(GS) + 4W^−1^ + e^−^, while after 2p ionization, the process is: Al^4+^(2p^−1^) + 2W → Al^3+^(GS) + 2W^−1^ + e^−^. Experimentally, we see that the ETMD feature after 2p ionization is shifted towards higher kinetic energy relative to the second ETMD step following 1s ionization. This can be qualitatively understood as being due to the differences in the amount of positively charged water ions around the Al atom in the final state. This is also consistent with the calculated maximum kinetic energies for the ETMD_2p−1→GS_ process after 2p ionization, being several electron volts higher than the estimated high-energy-tail energy of the second ETMD step following 1s ionization.

The previous discussions have been limited to ETMD(3) processes. The alternative ETMD(2) processes (Fig. 2) result in formation of H_2_O^2+^. This doubly charged water ion will most likely rapidly form H_3_O^+^ and OH^+^ via proton transfer to another water molecule^43^, thereby further contributing to the local formation of reactive species.

## Summary and outlook

X-ray absorption by solvated metal ions, multi-step electronic decay processes involving non-local ETMD steps, and the resulting radiation damage by extensive local water ionization have previously been discussed using Mg^2+^ in water as a theoretical example^5^. The predictions for Mg^2+^ could not be experimentally verified so far. However, for the experimentally favourable aqueous Al^3+^ system, we have observed the spectral signatures of consecutive ETMD processes using liquid-jet photoemission spectroscopy. Initially, the 1s levels of aqueous Al^3+^ ions were ionized with synchrotron radiation. Local KLL Auger–Meitner decay leads to the Al^5+^(2p^−2^) state (and, to lesser degrees, 2s^−1^2p^−1^ and 2s^−2^ states), which cannot relax by local Auger–Meitner electron emission. Instead, their electronic relaxation involves two successive ETMD steps. Both of these ETMD steps produce distinctive spectral features, which we could experimentally identify by their kinetic-energy profiles and comparison with the ETMD electron peaks associated with 2p-photoionized aqueous Al^3+^ ions.

The energy absorbed in the 1s-ionized aluminium ion is mainly dissipated by the outgoing Auger–Meitner electron, which may lead to additional ionization events beyond the first solvation shell of Al^3+^. However, substantial local ionization has also now been shown to occur. Our observation of two consecutive ETMD steps implies that each Al 1s ionization event results in up to four singly charged, neighbouring water ions. In addition, the low kinetic energies of the two ETMD electrons means that these electrons have particularly short inelastic-scattering mean free paths of ~1–2 nm in the surrounding water^44–46^. These electrons can potentially ionize five to six more water molecules, so a single Al 1s ionization event can, thus, result in up to ten ionized water molecules in the vicinity of the excitation, forming a local spot of cascaded radical chemistry and further radiation damage.

Standard models in radiation chemistry typically assume a random ionization probability along the track of the ionizing particle. The experimental verification of decay cascades involving multiple non-local autoionization events, as presented here, should lead to a revision of these models. The two-step ETMD process generates a large number of ionized water molecules and slow electrons at a single site and within a very short time. These ionized water molecules will develop into radicals—such as OH (ref. ^6^), on a timescale shorter than 1 ps—which will probably reactively combine to produce hydrogen peroxide, H_2_O_2_ (ref. ^47^). Furthermore, the slow electrons can thermalize and react further with hydrogen peroxide, producing an OH^−^ and OH pair. Additionally, the hydrated electron can also reduce the metal cation. Notably, such radiolytic processes may also be important in the field of astrochemistry, with potential contributions to the planetary budget of oxidative species^48^.

Sometimes, radiation damage is a desired effect. High-*Z* atoms can be excited resonantly, funnelling the X-ray photon energy into a specific atom and charge-multiplication processes. Schemes involving selective core-level excitation/ionization of metal nanoparticles or iodinated molecules, which act as so-called radiosensitizers, have been studied for potential applications in tumour treatment (Auger–Meitner therapies)^49–53^, as well as a potential tool for the defined transformations of materials, via X-ray photochemistry^54,55^. Cascaded ETMD processes, such as those experimentally confirmed here, may be utilized in the design of the associated X-ray radiosensitizers.

The aluminium ions studied here can be taken as models for various atoms with higher *Z* than the low-*Z* atoms that predominate in biomaterials. With increasing *Z*, deep-core ionization will, via Auger–Meitner cascades, produce ions of increasingly higher charge. For example, Ca and P are the two most abundant third-row elements in the human body, with ~1.4 and ~1.1 mass per cent, respectively^56^. After 1s ionization, the associated excited states will mainly decay via a three-step Auger–Meitner cascade, thereby emitting three more electrons. Filling these four electron vacancies by consecutive ETMD processes will result in up to eight ionized water molecules in the immediate vicinity of the absorber, plus a number of additional ionized molecules due to the emitted Auger–Meitner and ETMD electrons. We note that this is not limited to solvated ions, but similar processes will also take place upon ionization of metal atoms in, for example, metalloproteins, leading to extensive local damage to the molecular structure. After Ca and P, the main high-*Z* atoms in the human body are S, Na, K, Cl, Mg, and Fe. Such high-*Z* atoms will contribute more to local radiation damage than their concentration suggests, due to their higher ionization cross-sections at the photon energies of relevance for X-ray applications. Following an associated X-ray photoabsorption event and the subsequent local Auger–Meitner ionization cascade, the results reported here suggest that cascaded ETMD processes will occur and lead to charge multiplication in the solvation shell of the ionized centre. Importantly, these ETMD processes can be expected to lead to far greater degrees of local radiation damage than generally contemplated.

## Methods

### Experimental methods

**Extended Data Fig. 2:**
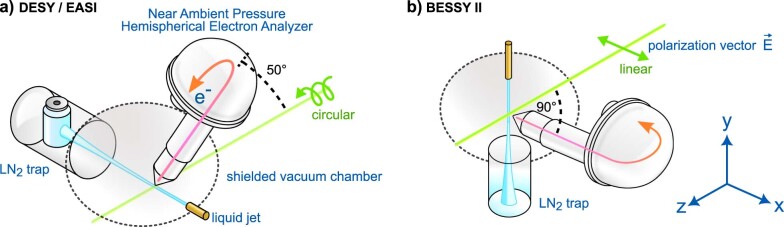
Sketches of the experimental setups. Setup at DESY (a) and BESSY II (b), showing the arrangements of the liquid jet, direction of the incoming photon beam (including the respective light polarization), and the direction of electron detection. (a) Electron detection is in backward direction with respect to the circularly polarized photon beam propagation. (b) Electron detection is parallel to the polarization vector of the linearly polarized light.

A liquid-microjet setup coupled with a hemispherical electron energy analyser was used to measure the slow electrons formed in the autoionization processes. The measurements of the ETMD electrons upon 1s and 2p ionization were carried out separately at two different beamlines. The 1s ionization experiments were performed at the P04 beamline of the synchrotron facility PETRA III, DESY, Hamburg^57^, using X-rays of ~1,570 eV photon energy, with circular polarization, and the Electronic structure of Aqueous Solutions and Interfaces (EASI) liquid-jet photoemission setup ^34,58^. The X-ray beam was perpendicularly incident on the laminar portion of the liquid microjet and the emitted electrons were collected at a detection angle of 130° with respect to the photon-beam propagation direction, with the analyser mounted in the vertical plane (backward-scattering geometry, see ref. ^58^ and Extended Data Fig. 2a). The electrons were thus collected in a near-magic angle configuration, minimizing any differential sensitivity to the electron angular distributions. The measurements of the ETMD processes after 2p ionization were performed using a photon energy of 210 eV at the no-longer-existent U41_PGM beamline at the BESSY II synchrotron facility in Berlin^59–61^. The associated experimental setup has been described previously^62^ and was similar to the one described above, except for a 0° detection angle, as illustrated in Extended Data Fig. 2b. These data were recorded with the electron-analyser collection axis aligned parallel to the polarization vector of the linearly polarized X-rays. All experiments were performed at an approximately 10 °C liquid-jet temperature. The jets were formed using quartz-glass capillaries with 28 and 25 μm inner diameters, at DESY and BESSY II, respectively. We note that, for the low-energy ETMD electrons detected here, the probing depth into the solution is of the order of 2–4 nm (ref. ^46^).

Commercially purchased AlCl_3_ (Sigma-Aldrich, >98% purity) was dissolved in MilliQ (18.2 MΩ cm^−1^) water to prepare 2.0 M or 1.5 M solutions. The speciation in the solution depends on pH and concentration, and for 2 M and pH ≤4 solutions, the aluminium ions are mainly found as the aluminium hexahydrate cation ([Al(H_2_O)_6_]^3+^), that is, Al^3+^ surrounded by six water molecules^63^. The aluminium chloride solutions used in the measurements had a pH <2, where the amount of Al^3+^–Cl^−^ contact ion pairs was negligible, as further corroborated by molecular dynamics simulations^34^.

The kinetic-energy region associated with the ETMD spectral contributions coincides with a high background signal of inelastically scattered electrons; therefore, a separate measurement of the scattering background was necessary to isolate the ETMD electron features. For this purpose, we used a 50 mM NaCl aqueous solution (green spectra in Fig. 4). Due to water’s low conductivity of less than 1 μS m^−1^, even the nominally neat water used in liquid-jet photoemission experiments must contain a small amount of electrolyte (usually 5–50 mM) to assure sufficient electrical conductivity, avoid sample charging under irradiation, and permit reliable measurements of electron kinetic energies^64^.

### Theoretical methods

The theoretical approach that was used to interpret the experimental ETMD spectra was based on a combination of nuclear-ensemble methods for sampling the configurational space and subsequent ab initio calculations for the associated ensemble of configurations. The classical dynamical simulations used for sampling were described in detail in ref. ^34^. The simulations were performed for 2.0 M AlCl_3_ solutions to match the experimental conditions. The molecular dynamics simulation served to generate structural snapshots (20 structures). For further calculations, we selected two cluster sizes: [Al(H_2_O)_4_]^3+^ and [Al(H_2_O)_6_]^3+^. In the case of [Al(H_2_O)_4_]^3+^, we included the four closest water molecules from the larger cluster, [Al(H_2_O)_6_]^3+^. We are fully aware that the smaller [Al(H_2_O)_4_]^3+^ model does not fully capture the coordination shell of the Al^3+^ cation. On the other hand, it provided us with a valid proxy and allowed us to control the wave function and its convergence. The quantum chemical calculations of the ETMD states were performed for the structural snapshots in a polarizable-continuum model, within the non-equilibrium formulation, to partially mimic the solvent effects^65,66^. It is important to mention that the final states are highly charged (up to 7+) and the solvent effects can be included only approximately. Especially the smaller cluster size ([Al(H_2_O)_4_]^3+^) cannot correctly capture the absolute energies of the ionized states; however, since we estimate the kinetic energies of the ETMD electrons as energy differences, the large associated errors are partially cancelled out on an absolute energy scale.

The binding energies of the core levels and lowest-energy final states associated with the first ETMD step were estimated using the MOM^67^. The energies of the final ETMD states were calculated as the difference between the ground electronic state of the cluster and the energy of the cluster with additional electron holes (considering various multiplicities of the final states). The MOM calculations were performed using the polarizable-continuum model at the DFT/LC-*ω*PBE level (the range-separated parameter *ω* was set to 0.4 Bohr^−1^) with the aug-cc-pCVTZ basis set for an aluminium cation and the aug-cc-PVTZ basis set for all other atoms. The higher-energy final states of the second ETMD step were also calculated at the CAS-CI level with the cc-PVDZ basis set. Since such simulations can only be performed for molecules in the vacuum and were performed at a different level of theory, the lowest CAS-CI energies were shifted to match the MOM energies of the same final states with the same multiplicity, corresponding to the second ETMD step, for example, the MOM energies represent the onset of the spectra. Note that direct calculations of the electron signal intensities are simply impossible for complexes with such complicated electronic structure, in such a complex environment. The MOM calculations were performed in the Q-Chem 5.4 code^68^, and the CAS-CI calculations were performed in the TeraChem, v1.9, code^68–70^.

## Online content

Any methods, additional references, Nature Portfolio reporting summaries, source data, extended data, supplementary information, acknowledgements, peer review information; details of author contributions and competing interests; and statements of data and code availability are available at 10.1038/s41557-023-01302-1.

### Supplementary information

Supplementary Information Supplementary discussion.

### Source data

Source Data Fig. 4 Combined experimental and theoretical data for reconstruction of Fig. 4.

Source Data Extended Data Fig./Table 1 Geometries of clusters used for ab initio calculations.

## Figures and Tables

**Extended Data Table 1:**
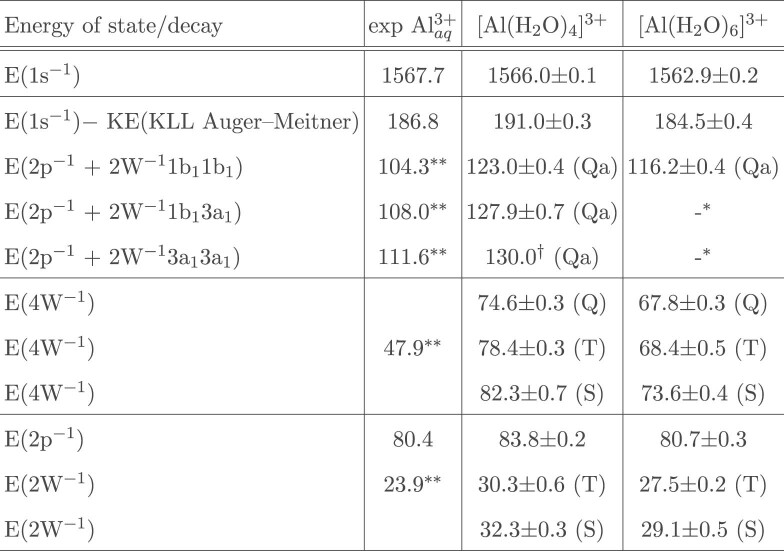
Energies of various charged states corresponding to the decay transitions

Average energies and mean-square deviations of the various charged states corresponding to the decay transitions. The calculations were performed for 20 clusters of [Al(H_2_O)_4_]^3+^ and [Al(H_2_O)_6_]^3+^ in a polarizable continuum at the MOM/LC-*ω*PBE level with the aug-cc-pVTZ basis set and aug-cc-pCVTZ on aluminium. Q (quintet), Qa (quartet), T (triplet), and S (singlet) denote multiplicities of the final states. **These values are estimated as the sum of the experimental binding energies of 2p^−1^ of aluminium and the calculated energies of a 1b_1_ or 3a_1_ electron of water next to an aluminium center, from Ref. ^34^ [E(2p^−1^) = 80.4 eV, E(1b_1_) = 11.97 eV, and E(3a_1_) = 15.6 eV] without the Coulomb penalty. *It was not possible to obtain the energy due to poor convergence of the wave function. †This value was obtained only for a single geometry due to poor convergence of the MOM wave function.

Source data

## Data Availability

Data relevant for this study are available at 10.5281/zenodo.7289021. Source data are provided with this paper.
