# Supplementary material for: Radiation damage by extensive local water ionization from two-step electron-transfer-mediated decay of solvated ions
Source: Nat Chem. 2023 Aug 24;15(10):1408–14. doi: 10.1038/s41557-023-01302-1 (PMC10533389; doi:10.1038/s41557-023-01302-1)
Supplement: Supplementary file 1 — Supplementary discussion. [file 41557_2023_1302_MOESM1_ESM.pdf]

nature chemistry

Article

https://doi.org/10.1038/s41557-023-01302-1

Radiation damage by extensive local water ionization from two-step electron-transfer-mediated decay of solvated ions

In the format provided by the authors and unedited

SUPPLEMENTARY INFORMATION

In this section, we provide a more detailed discussion of alternative decay cascades and ultrafast dynamics that can take place after 1s ionization and we aim at showing that the contribution to the measured signal is minor.

In a weakly bound $NeKr_2$ cluster, nuclear dynamics in the decaying $2p^{-2}$ state was found to have a significant impact on the final spectrum [1]. The shape of the potential energy surface in the $2p^{-2}$ state leads to a significant bond contraction supporting orbital overlap, which is then manifested by an exponential increase of ETMD intensity (and a change of kinetic energy of the outgoing ETMD electron to higher energy). In contrast, aluminium-water distances in the liquid phase are significantly shorter and the intermolecular bonds are stronger and directional (1.8 Å for Al–O). According to the MOM calculations for the smaller cluster size, the contraction of the Al–O bonds in the $2p^{-2}$ state is energetically unfavourable. Correspondingly, we can assume that the ETMD process is already fast in the initial geometry and that nuclear dynamics do not alter the measured spectra.

To complete the discussion of decay cascades following Al^{3+} 1s ionization, we must consider two more configurations of Al^{5+} formed in the Auger–Meitner decay step: $2s^{-2}$ and $2s^{-1}2p^{-1}$. These states are estimated to be reached with ∼6% and ∼21% probability, see Extended Data Figure 1. Due to the absence of 3s and 3p electrons in the aqueous Al^{3+} ions, regular Coster–Kronig decay of the configurations involving a 2s vacancy, as observed in metallic Al, cannot occur. Instead an ICD process may occur, in which a 2p electron fills the 2s vacancy and an electron from a neighboring water molecule is emitted: $Al^{5+}(2s^{-2})$ + W → $Al^{5+}(2s^{-1})(2p^{-1})$ + W^{-1} + e^-, or $Al^{5+}(2s^{-1})(2p^{-1})$ + W → $Al^{5+}(2p^{-2})$ + W^{-1} + e^-. The lifetime of this Coster–Kronig-type ICD has been shown to be very short, of the order of 1 fs for $2s^{-1}$ [2]. Thus, the ICD channel is much more efficient than the alternative ETMD pathway. A 2s vacancy created after the Auger–Meitner decay will therefore go through a fast intermolecular Coster–Kronig-type decay in one or two steps depending on if the configuration after the Auger–Meitner decay was $2s^{-1}2p^{-1}$ or $2s^{-2}$. The final result will be a $2p^{-2}$ configuration, which will then go through an ETMD cascade, similar to that described for the KLL Auger–Meitner decays with the same final-state configuration.

The energies of the electrons emitted in the aforementioned ICD processes can be estimated from the energy difference between the electronic states involved, the ionization

energy of the neighboring molecule, and the Coulomb penalty from the electrostatic repulsion between the ions, in the same way as described for the ETMD processes. The energies of the $2s^{-2}$ and $2s^{-1}2p^{-1}$ configurations for the aqueous Al^{5+} ions were not experimentally determined in these measurements, but the energy difference between the states due to these configurations and $2p^{-2}$ can be approximately estimated from data from metallic Al [3] or atomic Al^{5+} [4]. For atomic Al^{5+}, the energy difference between the various states with $2s^{-1}2p^{-1}$ and $2s^{-2}$ configurations lies in the range of 37.72 eV and 53.64 eV, and between the various states with $2p^{-2}$ and $2s^{-1}2p^{-1}$ configurations lies in the range of 29.11 eV and 55.97 eV [4]. In the experimental data on metallic Al of Dufour et al. [3], the observed energy difference between states with $2s^{-1}2p^{-1}$ and $2s^{-2}$ configurations lies in the range of 39 eV and 54.6 eV. The ^3P state of the $2p^{-2}$ configuration was not observed, but if the splitting between the $2p^{-2}$ $^3P_2$ and $2p^{-2}$ $^1D_2$ states is assumed to be the same as in atomic Al^{5+} (5.1 eV), the energy difference between states with $2p^{-2}$ and $2s^{-1}2p^{-1}$ configurations would lie in the range of 29.9 eV and 56.6 eV. The minimum energy required to ionize a neutral water molecule in the decay is $E(W^{-1})$ ~11 eV, and the lower limit of the Coulomb penalty energy E_{CP} is 0 eV. Relying on these estimates gives an upper limit of the kinetic energy of the electrons emitted in these ICD processes of ~45 eV. These decays can thus not explain the higher-energy feature in the Figure 4 top panel (a) nor the high-energy part of the low-energy feature, although they can be expected to contribute to the low-energy part of the low-energy feature to some degree.

Note that, for Auger–Meitner decays with one or two 2s vacancies, there will be one or two additional ionized water molecules in the vicinity of the Al ion and one or two more low-energy electrons compared to the situation after a KLL Auger–Meitner decay to a $2p^{-2}$ configuration, compounding the ionization-induced charge build-up and potential radiation damage.

---

[1] V. Stumpf, P. Kolorenč, K. Gokhberg, and L. S. Cederbaum. Efficient Pathway to Neutralization of Multiply Charged Ions Produced in Auger Processes. *Phys. Rev. Lett.*, 110:258302, 2013.

[2] G. Öhrwall, N. Ottosson, W. Pokapanich, S. Legendre, S. Svensson, and O. Björneholm. Charge

Dependence of Solvent-Mediated Intermolecular Coster-Kronig Decay Dynamics of Aqueous Ions. *J. Phys. Chem. B*, 114:17057–17061, 2010.

[3] G. Dufour, J.-M. Mariot, P.-E. Nilsson-Jatko, and R. C. Karnatak. K-LL Auger Spectrum of Aluminium. *Phys. Scr.*, 13:370–372, 1976.

[4] A. Kramida, Y. Ralchenko, J. Reader, and NIST ASD Team. NIST Atomic Spectra Database (Version 5.10), [Online]. Available: `https://physics.nist.gov/asd` [2023, March 23]. National Institute of Standards and Technology, Gaithersburg, MD 20899, 2022.
